# Coronavirus Disease 2019 (COVID-19) Manifestation as Acute Myocardial Infarction in a Young, Healthy Male

**DOI:** 10.1155/2020/8864985

**Published:** 2020-07-11

**Authors:** Prerak Juthani, Rohan Bhojwani, Neil Gupta

**Affiliations:** Yale University School of Medicine, 333 Cedar Street, New Haven, CT 06510, USA

## Abstract

Although a large part of the symptomology of coronavirus disease 2019 (COVID-19) has been attributed to its effects in the lungs, the virus has also been shown to cause extensive cardiovascular complications in a small subset of patients. In this case report, we describe a 29-year-old nonobese hospital food service associate who presented with diffuse abdominal and chest pain; he was found to be positive for severe acute respiratory syndrome coronavirus 2 (SARS-CoV-2) with significantly elevated levels of troponin T and multiple acute phase reactants; his EKG demonstrated ST-elevations consistent with anterolateral infarction. Despite having no significant past medical history or atherosclerotic risk factors, he was found to have a complete occlusion of his left anterior descending artery that required cardiac catheterization. This case demonstrates that cardiovascular complications must be considered in the COVID-19 population, even without the clear presence of other risk factors for heart disease.

## 1. Introduction

The first case of coronavirus disease 2019 (COVID-19) was reported in December 2019 in Wuhan, China [1]. Since then, the virus has spread across the globe, affecting over 9 million people and causing over 480,000 deaths. The pathogen behind COVID-19 is severe acute respiratory syndrome coronavirus 2 (SARS-CoV-2), an enveloped positive sense, single-stranded RNA virus. Majority of the patients infected with SARS-CoV-2 generally report respiratory symptoms, as the virus is known to cause inflammation by infecting type II pneumocytes by binding to the angiotensin-converting enzyme 2 (ACE2) receptor [2]. However, in a smaller subset of patients, SARS-CoV-2 has also been shown to cause cardiovascular complications [3]. Data regarding these patients have been less described. In this case report, we discuss a young patient who tested positive for SARS-CoV-2 and subsequently developed significant cardiovascular complications.

## 2. Case Presentation

**Figure 1:**
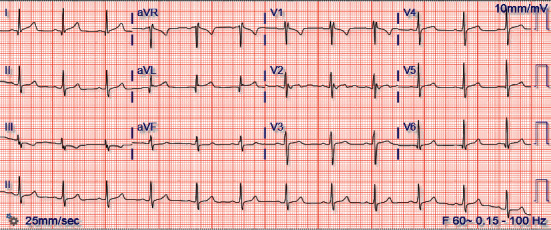
The patient's EKG on admission, which demonstrates sinus rhythm with nonspecific ST changes in the lateral leads alongside T-wave inversions in lead III. There were no observed ST-segment elevations or ST-segment depressions.

**Figure 2:**
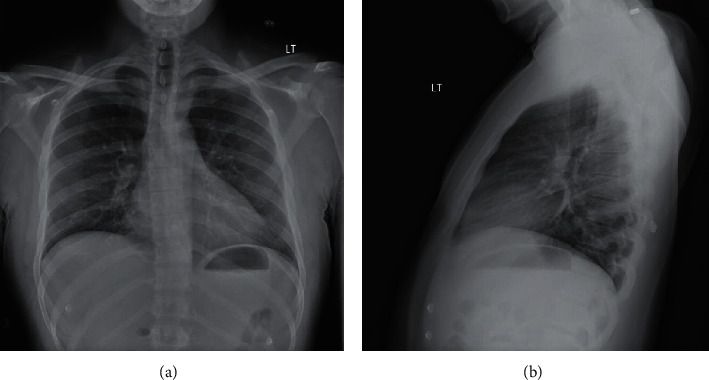
The patient's chest X-ray: (a) PA and (b) lateral. The lungs demonstrated no focal consolidation to suggest pneumonia and no evidence of pulmonary edema, pleural effusion, or pneumothorax. The cardiac silhouette was noted to be within normal limits, and no free air was detected below the diaphragms.

Our patient is a 29-year-old food service associate, working on the COVID-19 floor at a local hospital, who presented with diffuse abdominal and chest pain. He noted that his symptoms started the prior day when he had two bouts of emesis, after which he felt diffuse pain in his chest that persisted throughout the night. On presentation, the patient was a nonobese man (BMI: 26.4) who was afebrile (98.4°F), saturating well on room air (SpO_2_ = 100%) with a heart rate of 83 beats/min and a blood pressure of 142/81 mmHg. He denied any history of cough, shortness of breath, diarrhea, nasal congestion, or recent drug use. The patient was a nonsmoker, had no personal or family history of hypercoagulability, and had a slightly elevated LDL level (132 mg/dL), but no other atherosclerotic risk factors (HDL (49 mg/dL), cholesterol (195 mg/dL), triglycerides (70 mg/dL), and hemoglobin A1c (5.4%)). His family history was significant for a father who passed away from congestive heart failure (CHF) in his early 40s. Physical examination was unremarkable. The patient's initial lab reports demonstrated elevated troponin T (0.15 ng/mL), elevated AST (54 U/L), hyperkalemia (5.6 mmol/L), leukocytosis (12.9 x1000 cells/*μ*L), and thrombocytosis (492 x1000 cells/*μ*L). EKG revealed sinus rhythm with nonspecific ST changes in the lateral leads with T-wave inversions in lead III (Figure 1). A chest X-ray showed no evidence of focal consolidation, pulmonary edema, pleural effusion, or pneumothorax (Figure 2). Nasopharyngeal swabs were taken to assess for SARS-CoV-2 infection, and the patient was admitted to the floor.

**Figure 3:**
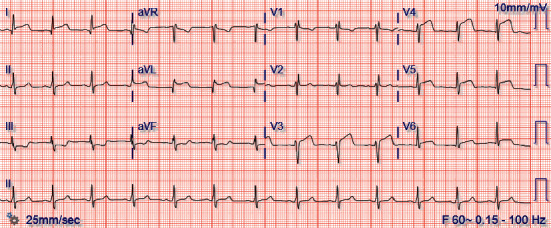
The patient's EKG on the morning of day 2. There were ST elevations in lead I, aVL, and the precordial leads overlying the anterior and lateral surfaces of the heart (V3–V6), which was consistent with anterolateral infarction. These findings were not present on admission (Figure 1).

**Figure 4:**
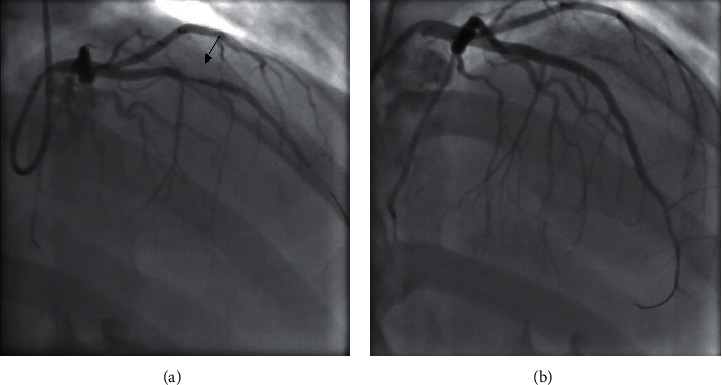
The patient's LAD thrombus (black arrow) (a) before and (b) after catheterization. Diagnostic angiography done prior to catheterization demonstrated complete midsegment occlusion of the LAD with no significant disease noted in the left main circumflex artery and the right coronary artery.

The patient was admitted for six total days. On day 1, the patient's results returned positive for SARS-CoV-2, and he was started on hydroxychloroquine (200 mg PO, twice daily). Multiple inflammatory markers were also elevated; these included fibrinogen (693 mg/dL), d-dimer (4.18 mg/L), ferritin (1,342 ng/mL), and CRP (149 mg/L). Of note, his procalcitonin was normal (2.2 mg/dL). The patient also had an elevated PT/INR (11.9 seconds, 1.15) and BNP (362 pg/mL). On day 2, the morning EKG revealed new anterolateral ST elevations (Figure 3) and transthoracic echocardiogram (TTE) demonstrated an ejection fraction of 35–40% with hypokinesis of the entire apex and the anterolateral segments, suggestive of an anterolateral myocardial infarction (MI). The patient was immediately taken to the cardiac catheterization lab, where he was found to have complete midsegment occlusion of the LAD (Figure 4), and a drug-eluting stent (DES) was placed. Given the patient's cardiac injury, hydroxychloroquine was discontinued and the patient was started on aspirin, atorvastatin, and prasugrel. Tocilizumab was considered, given the severe cardiovascular complications secondary to his SARS-CoV-2 infection, but was deferred, given its limited evidence in treating acute MI [4]. His clinical course after catheterization to discharge was uneventful with normal vital signs. On day 3, the patient was started on metoprolol, and on day 4, he was started on lisinopril. The patient was also started on enoxaparin because there were concerns of hypercoagulability in the setting of COVID-19 infection. Over the following two days, the patient reported resolution of his abdominal pain and chest tightness and had downtrending inflammatory markers with reassuring EKGs. He was discharged on day 6 with close hematology and cardiology follow-up.

## 3. Discussion

Severe acute respiratory syndrome coronavirus 2 (SARS-CoV-2) has created an enormous challenge throughout the world, given the complexity and unpredictable nature of its presentation. Though respiratory illness is thought to be the predominant manifestation for COVID-19 illness, COVID-19 is known to worsen cardiovascular disease and also create de novo cardiac complications [5]. According to the literature, the cardiac injury is thought to occur due to direct myocardial injury from viral involvement of cardiomyocytes, as well as systemic inflammation. In addition, systemic inflammation and shear stress from increased coronary blood flow are thought to increase the risk of plaque rupture as well as acute myocardial infarction [5]. The incidence of acute cardiac injury in COVID-19 patients has been estimated to be approximately 8–12% [6]. Despite these facts, studies have not described the incidence of ST-segment elevation myocardial infarction or the incidence of left ventricular systolic dysfunction in COVID-19 patients.

Our patient was a 29-year-old young male without significant medical comorbidities who presented to the hospital due to abdominal pain and chest tightness. He had no personal or family history of a hypercoagulability disorder and had no evidence of hypertension or diabetes, demonstrating very little evidence of underlying cardiovascular disease. He had stable vitals and a negative chest X-ray on presentation (Figure 2), with a mildly elevated troponin level and a highly elevated CRP level. Shortly following his admission, he developed ST elevations on his EKG with new left ventricular dysfunction on his echocardiogram. He ultimately required cardiac catheterization and placement of a DES stent in his LAD. We believe that this case is unique because this was a young, athletic patient with minimal risk factors for coronary disease who tested positive for COVID-19 and developed an acute MI with STEMI and required stent placement.

Though this patient did have mild hyperlipidemia (slightly elevated LDL (132 mg/dL)) and a family history of a father who passed away from CHF in his early 40s, we do not believe that these factors were enough to substantiate the significant coronary event he experienced. In fact, multiple studies have shown that, even though the atherosclerotic process begins at a young age in accordance with the level of traditional risk factors such as high cholesterol, clinical cardiovascular events do not occur until much later in life [7–9]. Furthermore, the Framingham Risk Score, a gender-specific algorithm that estimates the 10-year cardiovascular risk of an individual, does not even apply to patients under the age of 30 because it has been shown to overestimate the risk, even for those with risk factor burdens substantially greater than those of our patient [10]. For these reasons, we believe that the patient's cardiac pathology was a manifestation of his COVID-19 illness rather than his underlying risk factors.

In this particular case, the COVID-19 diagnosis also created unique challenges for the patient's cardiac care; all studies, including the echocardiogram and percutaneous coronary intervention, had to be done under nonideal circumstances (increased gowning, N-95 respirators, etc.) to minimize the risk of exposure to healthcare providers [11]. In addition, patient's age, underlying medical illnesses, and risk factors are all considered in triaging patients with COVID-19, as well as monitoring for respiratory and cardiovascular complications. In this case, the patient had no significant medical history and was athletic and healthy, and he developed evidence of myocardial infarction during his hospital course, with sudden left ventricular dysfunction of his heart, leading to the need for percutaneous coronary intervention. He was also started on anticoagulants, given the concerns of hypercoagulability with COVID-19, as COVID-19 infection has been thought to create a hypercoagulable state. The patient fortunately did well, and he was successfully discharged. It is a reminder to us that cardiovascular complications must be considered in the COVID-19 population, even in those patients with minimal risk factors for heart disease.

## Data Availability

The patient data used to support the findings of this study are restricted by the Yale Institutional Review Board in order to protect patient privacy. The data are available from Prerak Juthani for researchers who meet the criteria for access to confidential data.
